# Who is controlling whom? Reframing “meaningful human control” of AI systems in security

**DOI:** 10.1007/s10676-023-09686-x

**Published:** 2023-02-10

**Authors:** Markus Christen, Thomas Burri, Serhiy Kandul, Pascal Vörös

**Affiliations:** 1 Institute of Biomedical Ethics and History of Medicine, University of Zurich, Zurich, Switzerland; 2 Law School, University of St. Gallen, St. Gallen, Switzerland; 3 Armasuisse Science + Technology, Thun, Switzerland

**Keywords:** Autonomous systems, Meaningful human control, Security, Decision-making, Human-Machine-Interaction

## Abstract

Decisions in security contexts, including armed conflict, law enforcement, and disaster relief, often need to be taken under circumstances of limited information, stress, and time pressure. Since AI systems are capable of providing a certain amount of relief in such contexts, such systems will become increasingly important, be it as decision-support or decision-making systems. However, given that human life may be at stake in such situations, moral responsibility for such decisions should remain with humans. Hence the idea of “meaningful human control” of intelligent systems. In this opinion paper, we outline generic configurations of control of AI and we present an alternative to human control of AI, namely the inverse idea of having AI control humans, and we discuss the normative consequences of this alternative.

## Introduction

“Humans must retain control of AI and autonomous systems.” This imperative underlying political, ethical, and legal initiatives is particularly relevant when AI is used in security. AI is used for security purposes in the context of this opinion paper, when intelligent systems are deployed in military, law enforcement, emergency response, and rescue operations.

Actors in the security domain have clear motivations to involve systems that critically rely on machine intelligence (including Artificial Intelligence; AI) in order to operate autonomously. Decision-making in security contexts often happens under psychological stress, time pressure and uncertainty. It may be that there is insufficient information or, conversely, too much information to be processed in a short time. Furthermore, human security actors on the ground are often confronted with high personal risks, which may be reduced by using autonomous systems. For example, the use of bomb disposal robots has reduced the loss of human lives in the US Army in the wars in Iraq and Afghanistan (Singer, 2009). Furthermore, the military’s interest lies in reducing operating costs and personnel. Other drivers include the potential for force multiplication through multiple, low-cost systems and reduced dependence on communications links through increased autonomy of systems. However, for a system to act properly to fulfill those motivations, a minimal degree of autonomy will be required.^1^

Despite the advantages of autonomous systems in security, many challenges remain, e.g. how to create algorithms that equal human understanding of the world or reason about complex tasks, and to design versatile machines that can adapt to a wide variety of environments. To carry out missions in an armed conflict, intelligent systems must be able to recognize, classify, plan and decide on persons and objects while responding to threats in complex environments. To adapt to ongoing changes and be able to operate without human supervision, intelligent systems are in need of an increased capacity for learning. Here, technical and scientific developments have revealed a significant tension. Modern machine learning algorithms, especially from the field of Deep Neural Networks, impress with highly precise technical results. However, it is no longer guaranteed that machine learning processes and decisions can be understood because of the complexity of such deep learning systems, also known as black box systems. Since comprehensibility and traceability are essential features of scientific work, extensive research is done today to make AI explainable (Explainable AI). Understanding why and how machine learning algorithms come to certain output is also central for assessing legal, ethical, and economic risks.

So far, there is a consensus that humans should remain involved sufficiently in decision-making processes.^2^ This consensus rests also on the fact that decision-making in security involves human causalities in various ways: military operations involve killing of combatants and the risk of collateral damage, rescue operations may involve balancing risks for victims and rescuers or decisions on whom to rescue. Such high-stake decisions are embedded in deep cultural assumptions such as sacrificing human life for a greater good or guilt and shame when failing to save someone. When humans die in combat, policing or rescuing, we need to know whether their death was legitimate, who was responsible for it, and we may want to punish actors. We call this the “moral nature” of security decisions.

Intelligent systems risk falling short in addressing the moral nature of security decisions. They may, namely, be considered illegitimate killers breaking the martial law (Skerker et al., 2020), may not be able to explain why certain persons were targeted (Altmann, 2019), and there may not be a reasonable punishment for them (Sparrow, 2007).

Such considerations clarify why the notion of ‘meaningful human control’ was first developed with lethal autonomous weapon systems (LAWS) in mind. The term was minted within the Convention on Certain Conventional Weapons as a point of convergence to ensure that the use of autonomous weapons complies with basic principles of international humanitarian law; i.e. distinction, necessity, proportionality, and humanity (see Human Rights Watch, 2012; ICRC, 2018; Campaign to Stop Killer Robots, 2019). The notion of meaningful human control has been expanded beyond LAWS as part of various international initiatives aiming to establish governance with regard to AI and involving a broad range of actors: associations (e.g. IEEE, 2019), companies (Intel, 2017; IBM, 2018; Google, 2018), nongovernmental organizations (Asilomar, 2017), and international organizations (EU, 2019; OECD, 2019; Council of Europe, 2020; UN Secretary General, 2019; Winfield, 2019, alone listed 22 initiatives). Many of these initiatives discuss control of AI and autonomous systems as a key dimension of governance.

Despite this momentum to ensure human control, the precise implications of human control of AI and autonomous systems remain unclear and unexplored. What does ‘meaningful human control’ mean? When is an autonomous system effectively under such control? Serious doubts persist as to whether the term stands for substantive consensus regarding controlling LAWS at all (Crootof, 2016), is of a purely political nature (Marauhn, 2018), devoid of substance (Burri, 2019), or has been ill-defined (Canellas, 2015; compare with Green 2022). In response to such doubts, lawyers have attempted to fill the term ‘meaningful human control’ with substance (Scharre & Horowitz, 2015), while computer scientists have shown that all definitions so far proposed could still result in a mismatch between assigned functions and responsibility (Canellas, 2015).

On a more fundamental level, in both philosophy and law, control of one’s actions is considered a necessary requirement for responsibility (Morse, 1994; Fischer & Ravizza 2000). As Sparrow (2007) has argued forcefully, when a LAWS commits a war crime, it is not evident who should be held responsible, or whether anyone can be held responsible at all. The question whether such ‘responsibility gaps’ can arise has generated considerable debate (cf. Roff, 2014; Sassoli 2014). In international law, the existence of such gaps is relevant for both a humanitarian law that remains operable even with autonomous systems as well as state and individual criminal responsibility where the so-called standard of ‘effective control’ determines attribution (see ICJ Nicaragua v. US (1986), partly conflicting with ICTY Tadíc (1999)).

In this opinion paper, we refer to those discussions on meaningful human control and push our reflections on the notion as follows: First, we discuss basic configurations of control that involve both human and AI systems. Second, we outline some problems associated with the current focus on controlling AI systems (the “human oversight model”) that emerged in recent empirical studies we conducted. Third, we present an alternative model framed as “AI control of human deciders through consultancy with veto-power”. We then sketch some normative consequences of this model.

## Basic configurations of control

Before outlining basic configurations of controlling decision-making in security, we briefly sketch our understanding of ‘artificial intelligence’, since AI as a notion is notoriously fuzzy (for the notion of ‘autonomous system’, see footnote 1). Following a standard typology of computer science, ‘artificial intelligence’ refers to the attempt to replicate understanding and learning by means of an artifact, focusing primarily on either thought or action, as well as aiming for either a rational ideal or a replica of human capabilities (Russel & Norvig 2010).

This attempt usually begins with a choice which technology to use. ‘AI technology’ thus means the individual functions that can be implemented in computers to achieve artificial intelligence, e.g., a specific machine learning model. However, such algorithms by themselves are not operatively useful in decision-making contexts. They need to be implemented in an ‘AI system’, i.e., a structured, context-dependent combination of AI technologies for the purpose of achieving artificial intelligence. For example, a rescue robot equipped with various types of AI technologies – one for object recognition, one for gate control etc. – may constitute a system. Finally, we understand the term ‘AI decision’ as the conclusion of an AI system with real-world consequences.

It is important to note that AI decisions are never totally independent from human decision-making. Humans make design decisions on how to combine AI technologies to constitute an AI system; humans decide whether or not to use an AI system in a specified context; and humans interact with a system in various ways, for example, training it with data – i.e., choosing which data to use –, controlling some of the system’s operations, auditing and maintaining it, which in turn affects the decision-making capability of the system, etc. Thus, the idea of systems that are “fully autonomous” – let alone “superintelligent” – and thus entirely independent from human decision-making is beside the point. At least on a general level, humans have always been – and will always be – involved in the use of AI systems. Hence, in the following, our discussion is limited to the control of AI systems with regard to *mission-specific tasks* in security. This means, in particular, that neither design decisions, i.e., those concerning the construction of the system, nor strategic decisions, such as whether to use an AI system for a mission at all, form part of our considerations.

In the context of specified missions – e.g. the conquest of a strategically important position in combat or the recovery of people buried in a collapsed building – two levels of control can be distinguished (see also figure):^3^ On the *oversight level*, responsible actors with decision-making power (“commanders”) are in an position of oversight and decide upon the tactics to fulfill a mission. We explicitly limit the tasks to be fulfilled on the oversight level to defining and choosing options with a reasonable potential to contribute to fulfilling the mission; more general considerations, including, for instance, reflecting on whether a mission is reasonable at all, are not for the ‘oversight level’ to make.

The *operative level* concerns the execution of the tasks that are relevant and useful to fulfill the mission goal. On the operative level, various actors have to take various decisions and make use of tools for this purpose, which may be technologically sophisticated. When a drone is, for instance, used to map the situation in a disaster area autonomously, all decisions and tasks associated with the correct use of the drone, e.g. velocity, height, duration of use, would belong to the operative level. The decision to repeat a drone-based mapping to increase the probability of detecting victims, meanwhile, would be situated on the oversight level.

The distinction of these two levels is, of course, stylized. Real-world configurations are inevitably more complex and may require more than one hierarchical level of control.

**Fig. 1:**
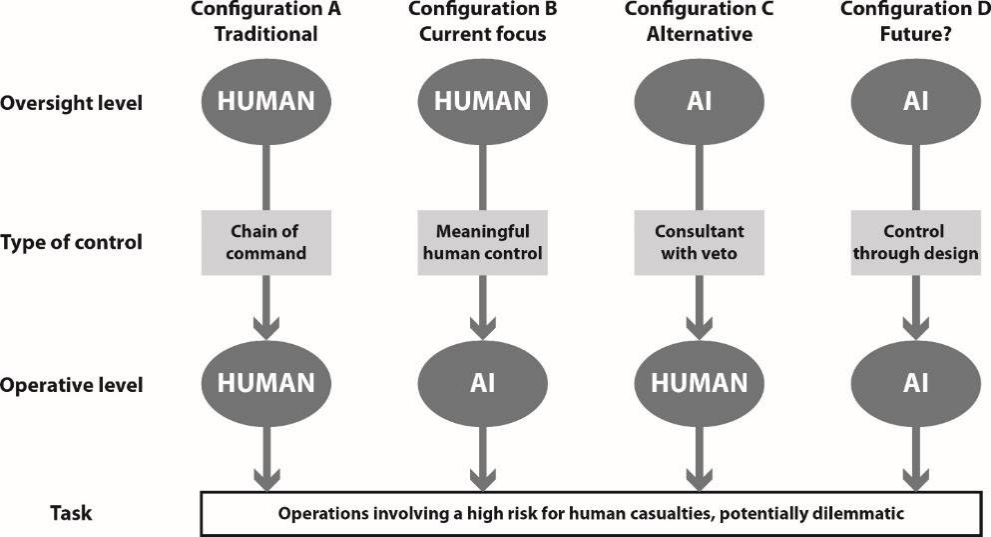
Outline of basic configurations of control

On both levels of control, the main actor can be either human or an AI system, leading to four possible configurations. In the traditional configuration (A) humans are present on both oversight and operative levels. The mode of control can be characterized as a “chain of command”. In security missions, it is usually clear who has which decision powers and responsibilities. For example, when a war crime was committed, the law draws a distinction based on whether the crime resulted from misuse or failure of command power or from failure of individual soldiers. The current debate about AI systems places them on the operative level (configuration B). In this configuration, the notion of ‘meaningful human control’ best captures the type of control. We will discuss this configuration in Sect. 3. In this paper, we propose a third possible configuration (C) where AI has the oversight function and “controls” the human decision-maker through the functions of consultancy and veto-power. The fourth configuration (D) where AI-systems control AI-systems on missions is by today’s standards a distant possibility which is beyond the scope of this paper.

## Challenges in controlling AI

Configuration B is best illustrated by means of a concrete vignette, outlining one of several possibilities of AI supervision^4^. We used the vignette for a study described below (Tolmeijer et al., 2022) in which an immediate threat arises due to a terror drone suspected of carrying a bomb towards a sports stadium and causing many casualties if reaching its target. A defense system is operating drones with net guns that can take down the terror drone. The defense system is equipped with AI tracks and predicts the terror drone’s flight path, positional data of the defense drones, data on urban population density and the like. As the terror drone is flying over populated areas, taking down the terror drone inevitably leads to collateral damage, as the bomb likely explodes as soon as the drone hits the ground. Based on the available data, the AI system calculates crash zones with expected low collateral damage and concludes where the terror drone should be taken down. There is human oversight, though, in the form of an operator receiving options for acceptable crash sites and conclusions from the AI system as to where to crash the drone. The operator is in a position to either confirm the AI’s conclusion with the chosen site or veto the AI’s conclusion and choose an alternative site. Thus, the operator supervises the system and has the possibility to intervene.

The vignette conveys a sense of how challenging it can be in practice to implement human control in configuration B. A surprisingly low number of *empirical* studies has investigated this. Since three of us co-authored a literature review on this (Kandul et al., submitted), we refrain from a deeper analysis for the purpose of this paper. We only outline some issues briefly that have arisen in two of our substantive studies.

Effective human control of AI relies on the ability of humans to predict AI “behavior”, e.g., recognize when AI is likely going to commit an error. Humans could then intervene and overrule the AI decision. In a laboratory experiment (Kandul et al., submitted), we asked participants to predict whether a specific operation AI is executing will be successful. The experiment involved a lunar lander game in which an AI navigated and landed a spaceship on a designated surface within a time limit. For the sake of the experiment, the operation was binary, resulting either in success or failure, i.e., safe landing or crash. We presented participants with a set of pre-recorded landings done either by an AI agent or a human expert operator. Participants watched the beginnings of a series of landings for a certain time and then predicted the success or failure of each landing based on the observed navigational pattern. Correct predictions resulted in an increased expected monetary reward for the experiment. Participants have correctly predicted the outcome of landings by the human operator about 10 p.p. more often than the outcome of landings by the AI agent (68% vs. 58% correct predictions, respectively). Moreover, participants took more time to make a prediction for the AI agent than for a human operator. This indicates that predicting the outcome of an AI landing was more difficult. When asked to guess the percentage of their correct predictions, participants failed to realize the difference in predictability of AI and human landings. We conclude that controlling AI in this context is more challenging than controlling humans due to lower predictability. The difference we observed might emerge in other settings where an AI’s approach to task solving is different from a human’s approach. Our results suggest that although a “human-AI” configuration (B) might be beneficial in that responsibility attribution seems clear, the overall performance might be improved with an “AI-human” configuration (C) where predictability of mistakes at the operational level potentially is higher.

In a further study on trust in AI in rescue and military contexts to which two of us contributed (Tolmeijer et al., 2022), we asked participants acting on the operative level to make a decision based on either an AI system’s or a human expert’s recommendation. Alternatively, on the supervision level, participants were asked to evaluate an AI system or a human expert decision and veto the decision in case they disagreed. The decisions involved choices among several possible options pertaining to the destinations of the drone, while the options differed in probabilities and the number of people affected by the decisions. The choices embodied an ethical dilemma, namely a tradeoff between the expected number of people who could be helped and the probability that at least some persons would be helped. Participants were put under time pressure. Two observations emerged from this study. First, the level of control – operational or supervisory control – does not seem to affect people’s level of trust in the expert. Participants equally trusted the recommendation and vetoed the decision made by others. Second, the AI system scored higher as an expert on a trust scale but lower on a moral responsibility scale. These results indicate that humans may be willing to delegate high-stake decisions to AI, as they believe that AI performs well in decisions in this context. However, humans believe that AI is less morally responsible than a human expert. This, in turn, indicates that the possibility to assign responsibility will also be relevant for the control configurations we discuss in this paper.

## What would be the implications of an AI “controlling” a human operator?

The results of our experiments draw the commonly accepted “human-AI” configuration (B) into doubt. First, it might be challenging for humans to exercise control of AI, especially in cases where AI predictability is low. Coming back to the motivations to include AI systems into security decisions, human control of AI may not decrease information management problems, as higher speed on the operative level implies less understandability. Second, if humans retain the power to take decisions, they could end up with the moral responsibility for the mistakes the AI committed. In other words, humans risk becoming “moral scapegoats” for mistakes operative AI systems committed. This could be considered the “moral price” to be paid for better decision making resulting from the configuration as a whole.

The two doubts just discussed usher in the configuration “AI-Human” (C). Concerning the first doubt, AI “control” of human actors also tends to address information management problems, while still increasing decision speed. As to the second doubt, since the moral dimension of decisions in security tends to manifest on the operative level where the sacrifice of human life occurs, the “AI-Human” configuration (C) where the human operator is retained as the main actor on the operative level offers a stronger coupling of the moral action with the moral agent, i.e. the human, than the inverse configuration (B). The risk that humans become “moral scapegoats” correspondingly decreases. The human acting on the operative level (C) is still able to exercise its freedom and act against the AI “controlling” it. In case of mission failure, the human actor will be rightfully blamed.

The vignette in Sect. 3 again serves as a good illustration. Configuration C would appear as follows in the context of the vignette. While the problem to solve remains the same, namely a terror drone menacing a sport stadium with collateral damage being unavoidable, a system is now in place in which an AI continues to serve for basic functionalities, namely, to inform the human operator but *not* to decide or recommend anything about the further course of action, e.g., predicting the flight path of the terror drone or identify and display crash sites. The decision where to go is up to the human operator who is in the position to fuse relevant sensor information. If the AI system detects indications for decision error due to operator stress or the operator indeed makes a clearly wrong decision, the AI system would intervene. Essentially, the main difference between configurations B and C is the sequence of decisions: In configuration B, the AI decides first, and the human has the possibility to change this decision – while the AI decision likely trumps the human judgment factually –; in configuration C the human decides first, and the AI has the possibility to alter this decision.

Let us analyze the configurations B and C under these premises in moral terms by assuming a “bad choice”. Under configuration B, the AI decides to crash the drone in the park because the available data indicates that this is not a heavily populated area and collateral damage, accordingly, is low. The human supervisor, preoccupied by the AI’s recommendation, does not check the specifics of the people in the park to be fast with the decision. Consequently, no intervention takes place. The choice turns out to be a bad one, though, because the local kindergarten happened to have its annual garden party in the park; although the number of persons in the park was indeed lower than at other crash places, many children died because of the AI decision. Consequently, the expectation is that the blame will not be assigned to the AI but rather the human supervisor who, however, could hardly have second-guessed the decision. In configuration C, in contrast, the human operator may base the decision on “human” perception by checking the specifics of the people and decide to bring down the drone somewhere else where more people are likely to die. The AI system would intervene and recommend the park – and now the human operator can finally decide and take the moral responsibility. Thus, in configuration C, a moral choice is made that is relatively clearly attributable to a human agent.

Before implementing the “AI-Human” configuration (C), several points need to be looked at closely. First, it must be ensured that AI performs well in “controlling” humans. AI must notably be able to predict human mistakes, at least on par with humans predicting humans’ mistakes. Second, protocols must be established for actions upon AI recommendation so that responsibility attribution is clarified. Third, one must ensure that AI supervision reduces psychological stress and uncertainty for the human actor. As the supervising AI generates additional input to the human actor and thus may increase time pressure, trade-offs with respect to mechanisms that decrease time pressure, e.g., because the AI is superior in identifying valuable options, need to be evaluated. Fourth, an AI veto power needs to be considered with a view to preventing obvious human errors. This, in turn, implies an answer to the question under which conditions humans may override an AI veto.

In sum, while changing the (theoretical) control configuration from “Human-AI” (B) to “AI-Human” (C) is unlikely to resolve all issues of complex human-AI interactions in security decisions, rethinking control in the light of the possibility of AI “controlling” humans (configuration C) may help us conceptually and lead to a sounder distribution of moral responsibility.

## Footnotes

1 We will not discuss the rich literature on machine autonomy for the purpose of this opinion paper. In the following, we understand ‘system autonomy’ (in AI/robotics) as a gradual concept that develops along several dimensions (Christen et al., 2017): Autarchy (the capacity of the system to function independently from external energy sources), independence from human control (i.e., the degree upon which the functioning of the system depends on human action or intervention), environment complexity, learning, and mobility.

2 For the purpose of this comment, we do not enter into the debate of taking humans completely “out of the loop” (see, e.g., Wagner, 2011).

3 Different terminologies exist to distinguish between the levels of control. In the military domain, Carl von Clausewitz (1832/2008; Chap. 3) introduced two levels: strategy and tactics. Aleksandr A. Svechin, an officer in the 1920s Soviet Red Army, first proposed the concept of an operational level (Kipp, 1992). Since 1982, the U.S. doctrine distinguishes between the strategic, operational, and tactical levels (de Czege & Holder, 1982). In the business context, however, it is more common to distinguish between strategic, tactical and operational decisions (Amason, 2001). As the term ‘strategic’ usually refers to high level decisions that go beyond specific missions and given that conflicting differentiations from the term ‘tactical’ exist, we use the terms ‘oversight level’ and ‘operative level’.

4 Two basic categories of AI supervision should be distinguished, namely, a first category in which AI systems perform mission tasks and human supervision involves controlling critical task decisions, whereas performing follow-up tasks *depends* on the tasks to be supervised, i.e., supervision occurs under time-pressure. In the second category, the task to be supervised and follow-up tasks are not dynamically connected, e.g., an AI system in border control deciding whether a person is allowed to cross the border. In the second category, the decision may be subject to supervision and can be revised. Our vignette falls into the first category where supervision is arguably more challenging.
